# How the Worm Turns, in Molecular Detail

**DOI:** 10.1371/journal.pbio.1001526

**Published:** 2013-04-02

**Authors:** Richard Robinson

**Affiliations:** Freelance Science Writer, Sherborn, Massachusetts, United States of America

Any behavior is, at its most elemental level, a coordinated series of muscle contractions. Those contractions are triggered by motor neurons, whose firing is controlled by other neurons, both excitatory and inhibitory, the whole chain set in motion by a stimulus. While the outlines of such a simple behavioral circuit have been understood for decades, the details—in particular, which neurotransmitters act through which receptors in which neurons—have remained largely unknown for even the simplest behaviors. In this issue of *PLOS Biology*, Jamie Donnelly, Chris Clark, Mark Alkema and colleagues tease out the details of one such behavior at the molecular level in the roundworm. They uncover a multi-step neurotransmitter cascade that allows a set of muscle cells to hypercontract on one side of the animal, causing it to turn sharply away from a threatening stimulus.

**Figure:**
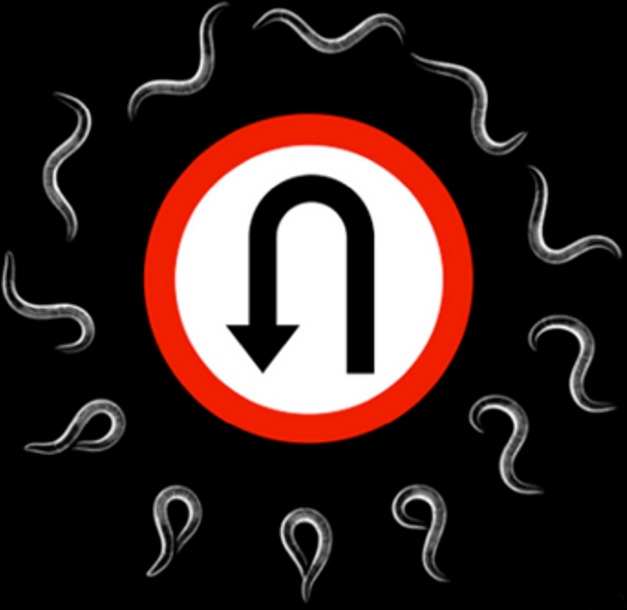
The nervous system of a roundworm orchestrates a series of muscle contractions that allow the animal to move backwards and turn away from a threatening stimulus. Image credits: Jeremy Florman, Chris Clark, and Mark Alkema.

Roundworms get about on their sides, alternately contracting and relaxing ventral and dorsal muscles to move sinusoidally through their environment. When a worm encounters a threat, it moves backward followed by a bend in the ventral direction (an “omega turn,” for the Greek letter it transiently resembles), and moves away in the opposite direction.

Tyramine, a monoamine neurotransmitter akin to noradrenalin, is known to be involved in the escape response in the worm. Earlier studies showed that a fast-acting tyramine-gated ion-channel controls the initial quick reversal of the escape. Here the authors found that a slow-acting G-protein–coupled receptor is required for the subsequent turn during the escape. When placed on a surface containing a high concentration of tyramine, worms become immobilized. In the present study, the authors found that this paralysis could be overcome by mutating the gene encoding the *ser-2* tyramine receptor. They also found another gene with the same effect, suggesting its involvement in the same pathway. This gene encodes a subunit of a so-called G protein, a well-known type of cellular signaling protein that inhibits neural transmission.

The authors found that *ser-2* was highly expressed in a set of neurons in the ventral nerve cord, in particular in 13 neurons (called VD neurons) that synapse onto ventral body wall muscles and release the inhibitory neurotransmitter GABA. Through a detailed set of experiments, they confirmed that tyramine-induced SER-2 signaling inhibited GABA release onto ventral wall muscles (i.e., reduced inhibition). Tyramine, therefore, promoted muscle contraction (and therefore the paralysis noted above was likely due to excess contraction, not lack of it).

In the next step, they used a laser to ablate VD neurons, and found that while the worm could still move sinusoidally, its ventral flexion was deeper than its dorsal flexion, leading it to turn in ventrally directed circles. Since GABA signaling from VD neurons sends an inhibitory signal, and since SER-2 inhibits this signaling, it seemed likely that SER-2, like neuronal ablation, could also induce ventral turning.

One possible way to test this hypothesis would be to switch the GABA neurons on and off directly, controlling the cells optogenetically. Optogenetics introduces two light-sensitive ion channels into neurons. Channelrhodopsin is activated by blue light, and serves to excite neurons and increase their firing. Halorhodopsin, activated by green light, does the opposite and serves to inhibit neuronal firing.

They found that blue light activation of GABA neurons that innervate the muscles on one side of the animal caused the worm to turn ventrally, while green light inhibition did the opposite, supporting the idea that GABA modulation can influence turning behavior. The remarkable effects of these opposing stimuli are preserved in a video of this remote control worm that accompanies the publication.

Finally, the authors showed that SER-2 regulates the tightness of the omega turn during an escape maneuver. When wild-type animals turn, the body bends so tightly in the ventral direction that the head makes contact with the body. But when SER-2 is missing, most bends are looser, with the head never touching the body as the worm changes direction. Without SER-2, they concluded, the GABAergic inhibition on VD neurons is incompletely shut down, preventing the hypercontraction of ventral wall muscles that allows the head to come round full circle.

Dissecting any behavior to this level of detail is an impressive feat of experimental technique, not to mention perseverance. This study provides an answer to both the simple question of how the worm turns, and the broader question of how a behavioral sequence is produced on the subcellular scale. Understanding a more complex behavior—such as catching a baseball —will require answering the same questions about the timing and location of neurons and neurotransmitters in the bewildering variety of circuits in our own nervous systems.

**Donnelly JL, Clark CM, Leifer AM, Pirri JK, Haburcak M, et al. (2013) Monoaminergic Orchestration of Motor Programs in a Complex** ***C. elegans*** **Behavior. doi:10.1371/journal.pbio.1001529**

